# Application of SGLT2 inhibitors in kidney diseases: a bibliometric analysis

**DOI:** 10.3389/fmed.2026.1768225

**Published:** 2026-02-06

**Authors:** Qin Hu, Hua Jin, Xu Li, Lan Hu

**Affiliations:** 1 The First Affiliated Hospital of Anhui University of Chinese Medicine, Hefei, China; 2 Department of Nephrology, The First Affiliated Hospital of Anhui University of Chinese Medicine, Hefei, Anhui, China; 3 Center for Xin’an Medicine and Modernization of Traditional Chinese Medicine of IHM, The First Affiliated Hospital of Anhui University of Chinese Medicine, Hefei, Anhui, China

**Keywords:** bibliometric analysis, CiteSpace, kidney disease, SGLT2 inhibitor, VOSviewer

## Abstract

**Purpose:**

Kidney disease represents a significant public health burden. Sodium-glucose co-transporter 2 (SGLT2) inhibitors, initially developed as antihyperglycemic agents, have demonstrated substantial renoprotective effects and emerged as a focal point in kidney disease research. This study aims to conduct a bibliometric analysis to evaluate the current state, research priorities, and evolving trends in SGLT2 inhibitor studies within the context of kidney disease.

**Methods:**

Relevant literature on “SGLT2 inhibitors” and “kidney diseases” was extracted from the Web of Science Core Collection, and the PubMed database was used to search for and supplement clinical research trend analysis. Bibliometric analyses and scientific visualizations were performed using R-bibliometrix, CiteSpace, and VOSviewer software to map the global research landscape.

**Results:**

A total of 1,561 publications published between 2009 and 2025 were analyzed. The annual number of publications has increased steadily. The United States is a core contributing country in this field, with strong international collaboration networks. Key institutional contributors include the University of Toronto and the University of Groningen, while prolific authors like Heerspink HJL and Wheeler DC are key contributors in this domain. Core journals are primarily concentrated in the domains of diabetes, nephrology, cardiovascular medicine, and metabolism, with the *New England Journal of Medicine* exerting the greatest influence. Research focus has evolved from glycemic control to multi-organ protective effects, with study populations expanding to include patients with non-diabetic CKD. Mechanistic investigations now extend to pathways involving inflammation, oxidative stress, and ferroptosis.

**Conclusion:**

SGLT2 inhibitors hold considerable promise in the management of kidney disease. Future research directions are expected to emphasize elucidating their underlying mechanisms, evaluating long-term safety, and developing personalized treatment strategies.

## Introduction

Kidney disease has emerged as a growing global public health challenge, attracting increasing attention in medical research due to its high prevalence and complex pathophysiological mechanisms (1). According to the latest data from the International Society of Nephrology (ISN), over 850 million individuals worldwide suffer from chronic kidney disease (CKD) at various stages, frequently accompanied by cardiovascular complications that significantly threaten patient morbidity and mortality (2). Diabetic kidney disease (DKD), a common microvascular complication of diabetes mellitus, affects an estimated 20 to 40% of diabetic patients and serves as the primary etiology of end-stage renal disease (ESRD) (3). Current standard therapies for CKD primarily rely on angiotensin-converting enzyme inhibitor (ACEI) or angiotensin II receptor blockers (ARB), which can slow disease progression. However, a substantial proportion of patients continue to progress to ESRD despite such treatment (4).

Sodium-glucose co-transporter 2 (SGLT2) inhibitors can lower blood glucose by inhibiting glucose reabsorption in the renal proximal tubules, thereby increasing urinary glucose excretion. They were initially used as hypoglycemic drugs for the treatment of type 2 diabetes mellitus (T2DM) (5). Conditions such as hypertension, diabetes, and glomerular diseases may induce glomerular hyperfiltration and renal hypoxia, resulting in increased proteinuria and progressive renal dysfunction. This pathological cascade heightens the risk of kidney disease and may ultimately lead to ESRD (6, 7). SGLT2 inhibitors mitigate these effects by restoring tubulo-glomerular feedback, thereby reducing glomerular hyperfiltration and intraglomerular pressure. Furthermore, by inhibiting sodium and glucose reabsorption, they decrease ATP consumption, alleviate renal hypoxia, and reduce metabolic stress on the kidneys (8, 9). In recent years, multiple large-scale clinical trials have demonstrated that SGLT2 inhibitors not only improve glycemic control in diabetic patients but also significantly reduce the risks of renal function decline, progression to ESRD, and major cardiovascular events in patients with CKD. Importantly, these renoprotective benefits appear to be independent of their glucose-lowering effects (10–12). As research advances, the therapeutic applications of SGLT2 inhibitors have expanded beyond diabetic nephropathy to include acute kidney injury (AKI) and non-diabetic CKD. Concurrently, significant progress has been made in elucidating underlying mechanisms, including modulation of tubulo-glomerular feedback, renal energy metabolism, and autophagy regulation (13–15).

With growing scientific interest, the body of evidence on the renal protective effects, efficacy, and safety of SGLT2 inhibitors has expanded rapidly. Although several systematic reviews have summarized aspects of SGLT2 inhibitor use in kidney disease, there remains a notable gap in bibliometric studies that systematically analyze and visualize the global research output in this domain. Bibliometrics enables quantitative assessment of the temporal and spatial distribution of publications, knowledge structures, and collaboration networks, offering an objective means to map disciplinary development, identify research hotspots, and forecast emerging trends (16). Unlike traditional reviews, bibliometric analysis not only traces the evolutionary trajectory and core themes of a field but also provides empirical data to guide researchers and policymakers in optimizing resource allocation and prioritizing future research directions.

This study comprehensively map the global research landscape of SGLT2 inhibitors and kidney disease through in-depth mining of relevant literature in the Web of Science Core Collection (WoSCC). Specifically, it examines the temporal and geographic patterns of scholarly output, identifies key contributors and institutional collaborations across countries and regions, and analyzes prevailing research themes and emerging frontiers. The findings will help researchers quickly grasp the current state and developmental trends in this field, enhance understanding of its intellectual evolution, inform future research and clinical practice, and support targeted investigations to advance both basic and translational knowledge.

## Materials and methods

### Data sources and search strategies

**Figure 1:**
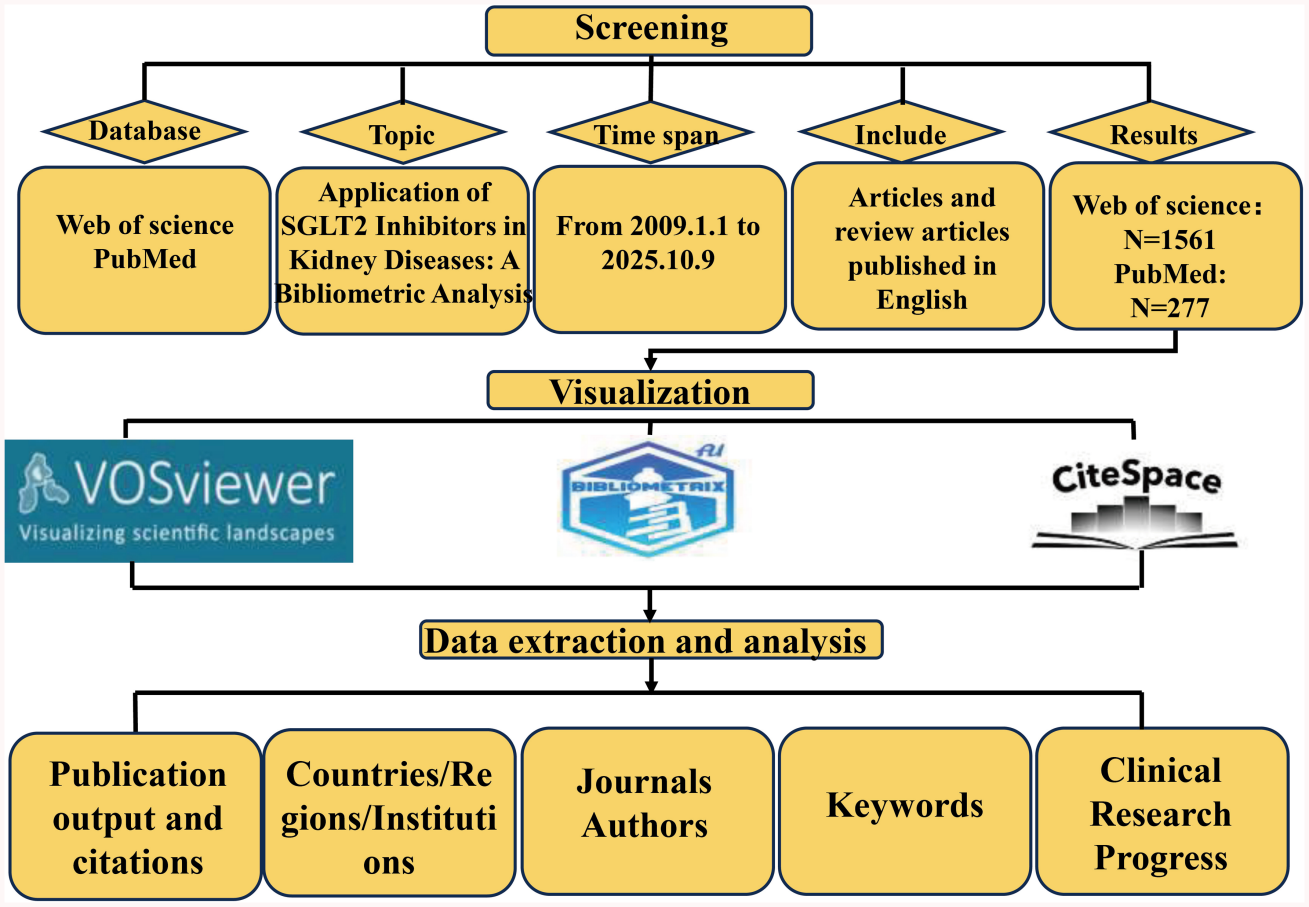
The flow chart of bibliometric analysis.

This study used the Web of Science Core Collection (WoSCC) as the data source for literature retrieval, specifically selecting the Science Citation Index Expanded (SCI-Expanded) and Social Sciences Citation Index (SSCI). The search strategy incorporated the keywords “SGLT2 inhibitors” and “kidney disease,” limited to English-language articles and review articles. The publication period was defined from January 1, 2009, to October 9, 2025. To mitigate the limitations arising from reliance on a single database, PubMed was incorporated as a supplementary source. The search strategy is detailed in the Supplementary material, and only 294 articles classified as “Clinical Trial” were selected to enhance the depth of analysis regarding trends in clinical research. EndNote (version 21.2) was employed as a reference management tool to identify and remove duplicate records across the Web of Science and PubMed databases. To ensure data accuracy and relevance, two independent researchers conducted screening by removing duplicates and studies unrelated to kidney disease, ultimately retaining 1,561 articles for analysis (Figure 1).

### Statistical analysis and visualization

Bibliometrics offers a systematic and quantitative approach to mapping the evolution of academic disciplines through the analysis of publication patterns, structural relationships, and thematic distributions. In this study, bibliometric analyses were performed using CiteSpace (6.3.R2), VOSviewer (1.6.20), and the Bibliometrix package in R (version 4.3.1). CiteSpace was employed to detect research hotspots and emerging trends via keyword co-occurrence and burst detection analyses, as well as to construct a timeline visualization of author keywords. Frequency-based descriptive statistics for authors, journals, institutions, countries, and keywords were generated using the Bibliometrix package. VOSviewer was utilized to visualize co-authorship relationships among countries/regions, institutions, and authors, as well as keyword co-occurrence patterns.

## Result

This study conducted a bibliometric analysis based on the Web of Science and PubMed databases, focusing on key areas according to each database’s strengths. The Web of Science dataset, comprising 1,561 valid publications, served as the primary data set for analyzing publication volume, countries, institutions, authors, journals, and keywords. The PubMed database included 277 publications, which were utilized to focus specifically on clinical research progress.

### Annual publication volume and trend

**Figure 2:**
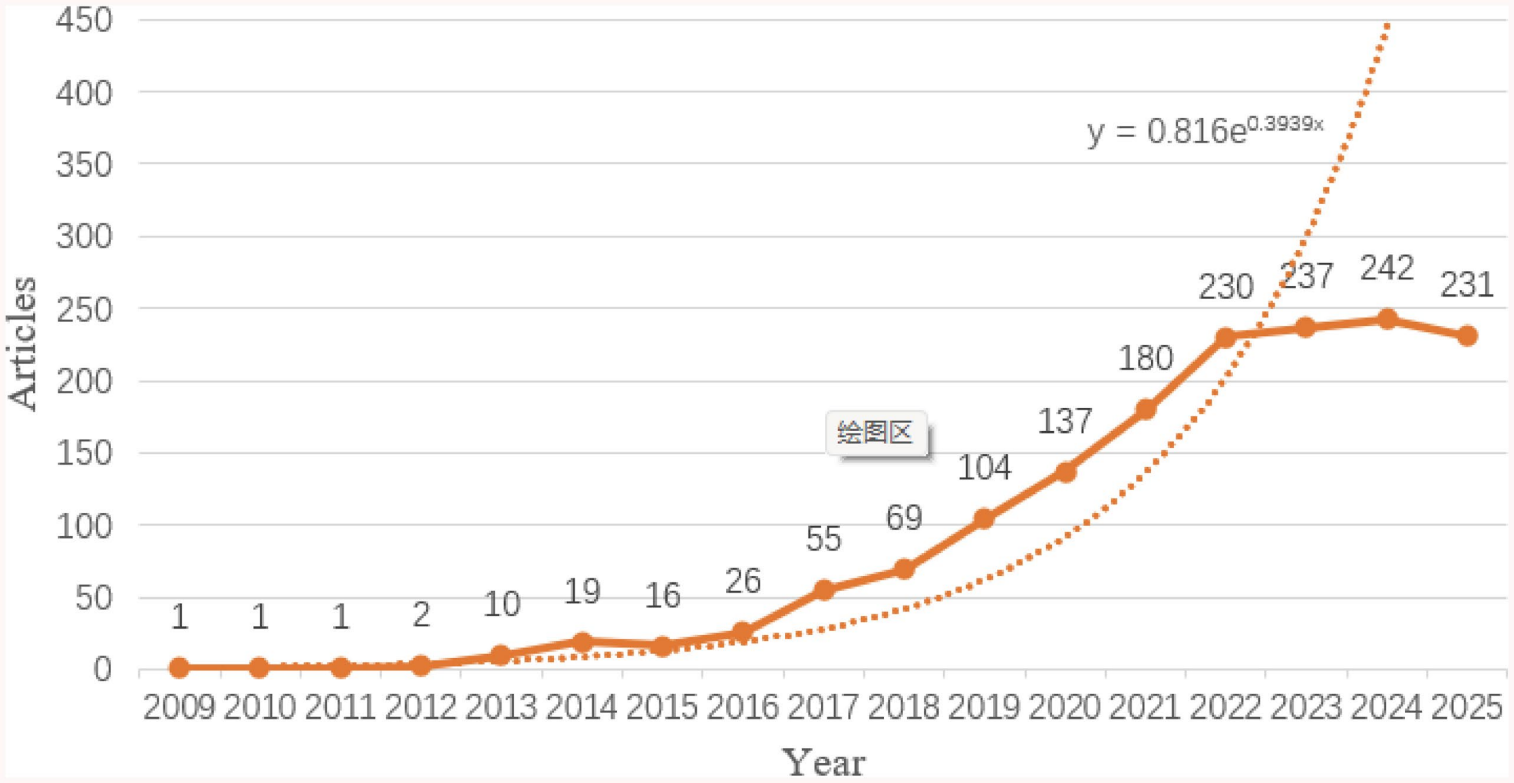
Annual publication volume and annual growth trend fitting curve.

A total of 1,561 valid publications were included in this study, comprising 1,103 articles and 458 review articles. Figure 2 illustrates the annual publication trend of SGLT2 inhibitor research in nephrology. Overall, the number of publications increased steadily from 2009 to 2025 and can be divided into three distinct phases. Phase I (2009–2015): Annual output remained low with slow growth, reflecting an exploratory stage of research. Phase II (2016–2021): A marked and sustained increase in publications was observed, culminating in 180 articles in 2021—11 times the volume published in 2015. This surge coincided with pivotal clinical trials such as EMPA-REG OUTCOME (12), CREDENCE (10), and DAPA-CKD (11), which demonstrated the renal benefits of SGLT2 inhibitors and catalyzed widespread interest in the field. Phase III (2022–2025): Publication output stabilized, with annual numbers consistently exceeding 230 articles and reaching a peak of 242 in 2024. As of October 9, 2025, 231 articles had been published. Although this figure does not yet represent a full year’s output, it aligns closely with previous annual totals, indicating ongoing high-level research activity and sustained scholarly engagement in the role of SGLT2 inhibitors in kidney disease (Figure 2).

### Analysis of countries and institutions

**Figure 3:**
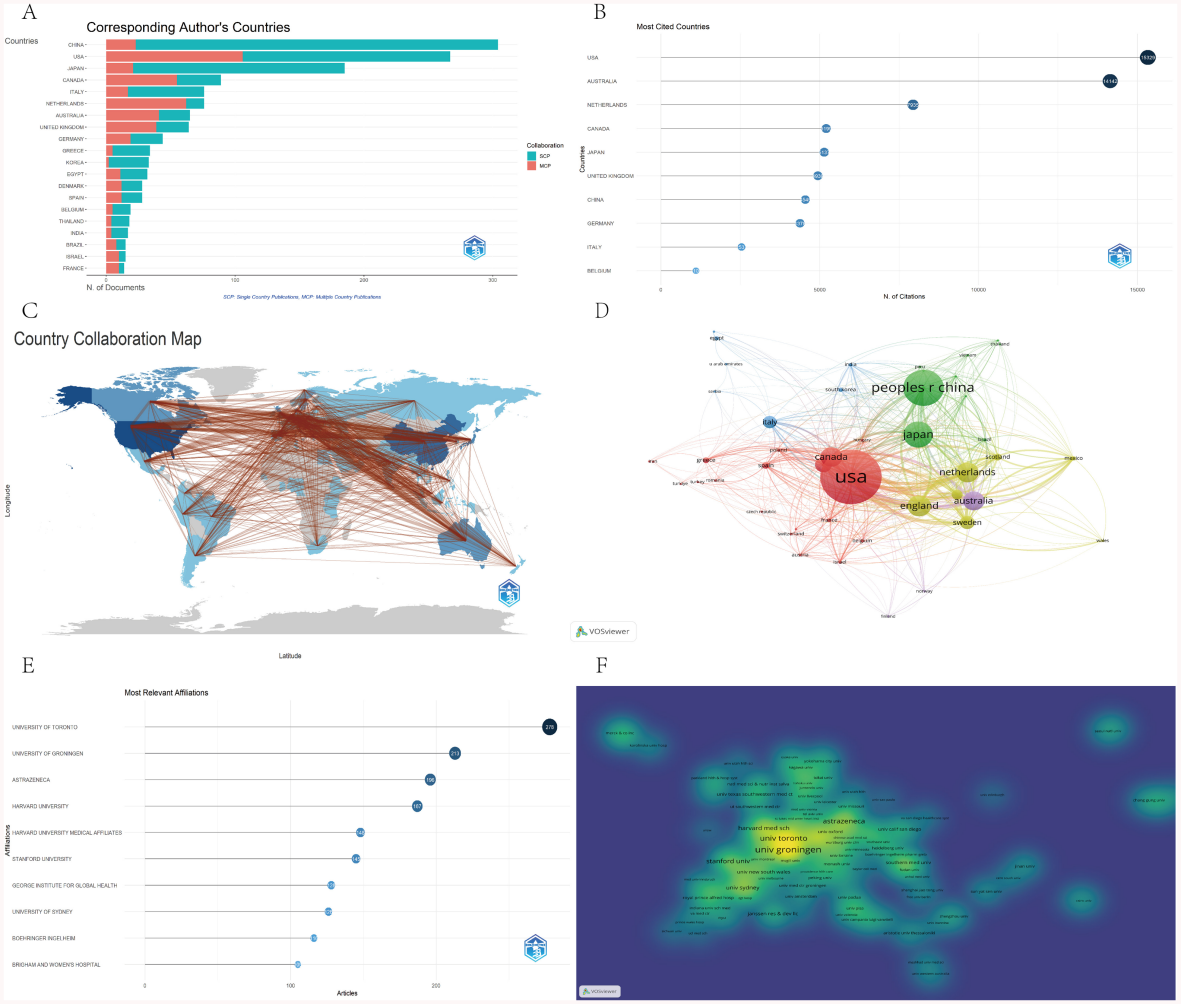
**(A)** Top 20 productive countries. **(B)** Top 10 most cited countries. **(C)** Country collaboration map. **(D)** Countries contribution and collaboration. **(E)** Top 10 most relevant affiliations. **(F)** Institutions contribution and collaboration.

Table 1 summarizes key indicators across three dimensions—research output scale, collaboration patterns, and academic influence—providing a comprehensive overview of global disparities in SGLT2 inhibitor research for kidney disease. Based on the “all authors’ countries” metric, the United States leads in total publications with 2,230 papers, followed by China (1,240 papers) and Japan (843 papers), reflecting international research collaboration networks. However, when ranked by the number of “corresponding authors’ countries,” China tops the list with 304 publications (19.5% of the total), indicating its central role in this field of research. The “corresponding author country” dimension is more suitable for reflecting the dominant nation in research. Although U.S. corresponding authors contributed 267 papers, their share of multiple country publications (MCP) reached 39.7%, significantly higher than China’s 7.6%, underscoring the U.S.’s stronger engagement in international collaborative research (Figure 3A). Citation metrics reveal further insights into research impact (Table 1). Despite having fewer total citations (14,142) than the United States (15,329), Australia exhibits a substantially higher average citation per article (217.60 vs. 57.40), suggesting greater per-paper influence. The countries with higher citation counts are all developed countries. China’s total citations amount to 4,545, with an average of only 15.00 citations per paper (Figure 3B). Figure 3C displays the international collaboration network, where the top 10 collaborating countries are predominantly high-income nations, including the USA, United Kingdom, Netherlands, Australia, and Canada. The most frequent bilateral collaborations are USA–United Kingdom (151 instances), USA–Netherlands (137), and USA–Australia (124), demonstrating the central role of the United States in shaping global research partnerships. As visualized in Figure 3D using VOSviewer, the thickness of connecting lines corresponds to the intensity of collaborative efforts between countries.

**Table 1:** Comparison table of national scientific research competitiveness indicators.

Country	Articles	Corresponding author articles	MCP (%)	Citations	Average article citations
USA	2,230	267	39.7	15,329	57.4
China	1,240	304	7.6	4,545	15
Japan	843	185	11.4	5,137	27.8
United Kingdom	511	64	60.9	4,939	77.2
Canada	484	89	61.8	5,199	58.4
Australia	484	65	63.1	14,142	217.6
Italy	437	76	22.4	2,537	33.4
Germany	392	44	43.2	4,378	99.5
Netherlands	391	76	81.6	7,935	104.4
Korea	193	33	6.1	887	26.9

Globally, 1,555 institutions contributed to publications in this field. As shown in Figure 3E, seven of the top 10 institutions by publication count are universities or affiliated academic centers. The University of Toronto (278 papers) and the University of Groningen (213 papers) rank first and second, respectively, representing the most productive institutional contributors. Notably, two multinational pharmaceutical companies—AstraZeneca (196 papers) and Boehringer Ingelheim (116 papers)—rank within the top 10, highlighting the substantial involvement of industry in advancing research on SGLT2 inhibitors. Figure 3F identifies institutions with the highest academic influence based on citation impact.

### Contributions of authors

**Figure 4:**
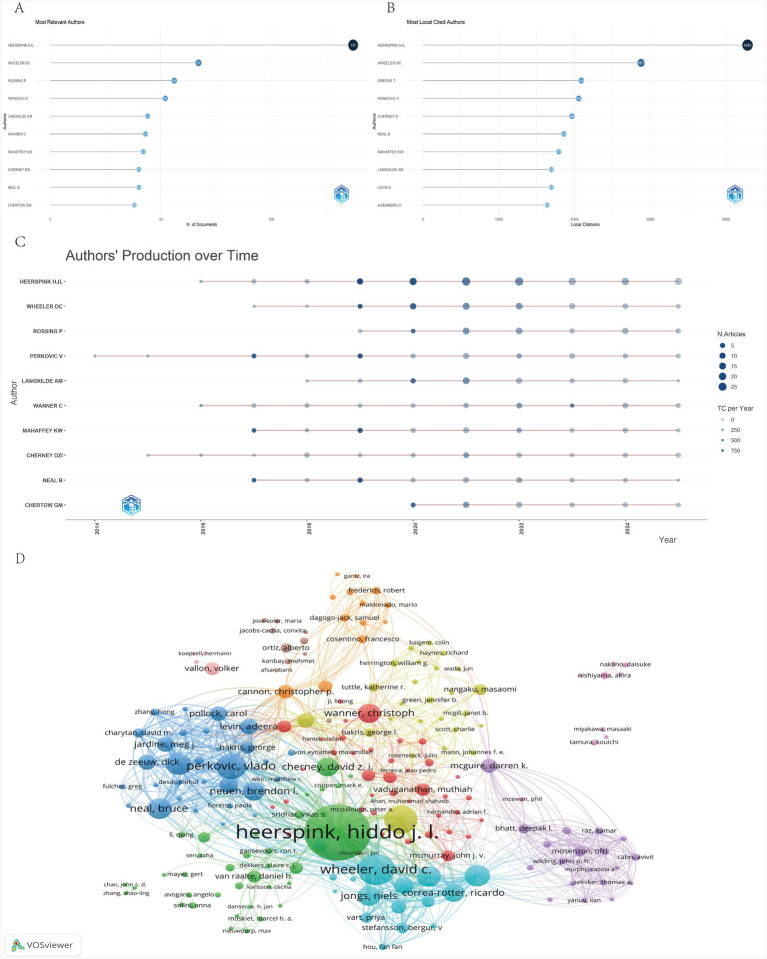
**(A)** Top 10 relevant authors. **(B)** Top 10 most cited authors. **(C)** Top 10 relevant authors’ production over time. **(D)** Author co-occurrence network.

A total of 8,912 authors have contributed to SGLT2 inhibitor research in nephrology. Figure 4A lists the 10 most prolific authors, who collectively published 527 articles—approximately 33.76% of the total corpus. Heerspink HJL from the University of Groningen authored 137 papers (8.78% of the total), making him the leading contributor in terms of output. Wheeler DC follows with 67 publications. As illustrated in Figure 4B, these high-output authors also rank at the top in citation frequency, reflecting both productivity and high scholarly recognition. The “authors’ production over time” plot (Figure 4C) traces the longitudinal research activity of the top 10 authors, revealing distinct publication trajectories. Heerspink HJL, the most influential author in the field, maintained consistent annual output from 2016 to 2025, with citation peaks observed in 2019. Author co-occurrence analysis (Figure 4D) effectively captures individual contributions and collaborative dynamics, where node size represents publication volume and connecting lines indicate co-authorship frequency.

### Core journals analysis

**Figure 5:**
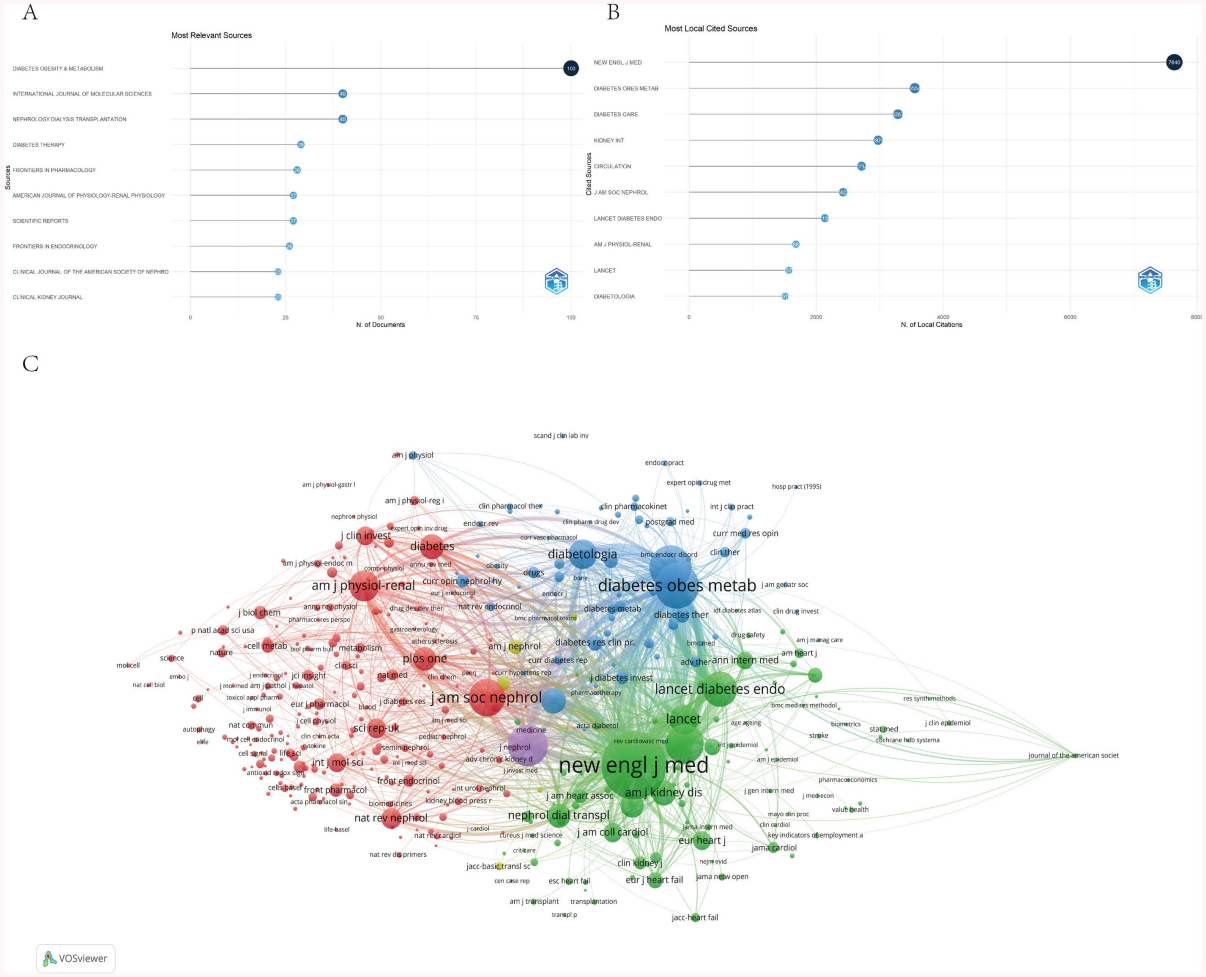
**(A)** Top 10 relevant sources. **(B)** Top 10 most cited sources. **(C)** Journals contribution and collaboration.

An analysis of the top 10 journals publishing research on SGLT2 inhibitors in kidney disease revealed that they collectively accounted for 23.25% of all publications, with a total of 363 articles (Figure 5A). *Diabetes, Obesity & Metabolism* emerged as the most active journal in this field, contributing 100 publications. Figure 5B presents journal citation frequencies, showing that the *New England Journal of Medicine* received the highest number of local citations (7,640), reflecting its prominent influence and high recognition within the research community. The H-index, which requires both substantial publication output and consistent citation impact, serves as a robust metric for assessing journal-level academic influence. Table 2 summarizes key indicators of journal impact, indicating that research in this domain is primarily disseminated through three thematic clusters of journals: diabetes-focused journals (*Diabetes, Obesity & Metabolism, Diabetes Care*); nephrology-oriented journals (*Kidney International, Clinical Journal of the American Society of Nephrology*); and those centered on cardiovascular and metabolic diseases (*Cardiovascular Diabetology, Circulation*). This distribution highlights the interdisciplinary nature of SGLT2 inhibitor research and provides guidance for researchers in selecting appropriate publication venues. The VOSviewer-generated co-citation network further confirms the central role of leading journals, with *New England Journal of Medicine, Diabetes, Obesity & Metabolism, and Diabetes Care* exhibiting the strongest citation linkages (Figure 5C).

**Table 2:** Journal academic impact.

Journal	H_index	Related articles	Citation	PY_start	Impact factor	JCR Partition
Diabetes Obesity & Metabolism	34	100	3,554	2013	5.7	Q1
International Journal of Molecular Sciences	19	40	640	2017	4.9	Q2
Nephrology Dialysis Transplantation	19	40	1,044	2015	5.6	Q1
American Journal of Physiology-Renal Physiology	18	27	1,681	2014	3.4	Q2
Kidney International	17	22	2,974	2009	12.6	Q1
Lancet Diabetes & Endocrinology	17	17	2,136	2014	41.8	Q1
Cardiovascular Diabetology	16	21	1,175	2017	10.6	Q1
Circulation	16	18	2,714	2016	38.6	Q1
Diabetes Care	16	19	3,287	2013	16.6	Q1
Clinical Journal of the American Society of Nephrology	14	23	1,035	2017	7.1	Q1

### Analysis of keywords

**Figure 6:**
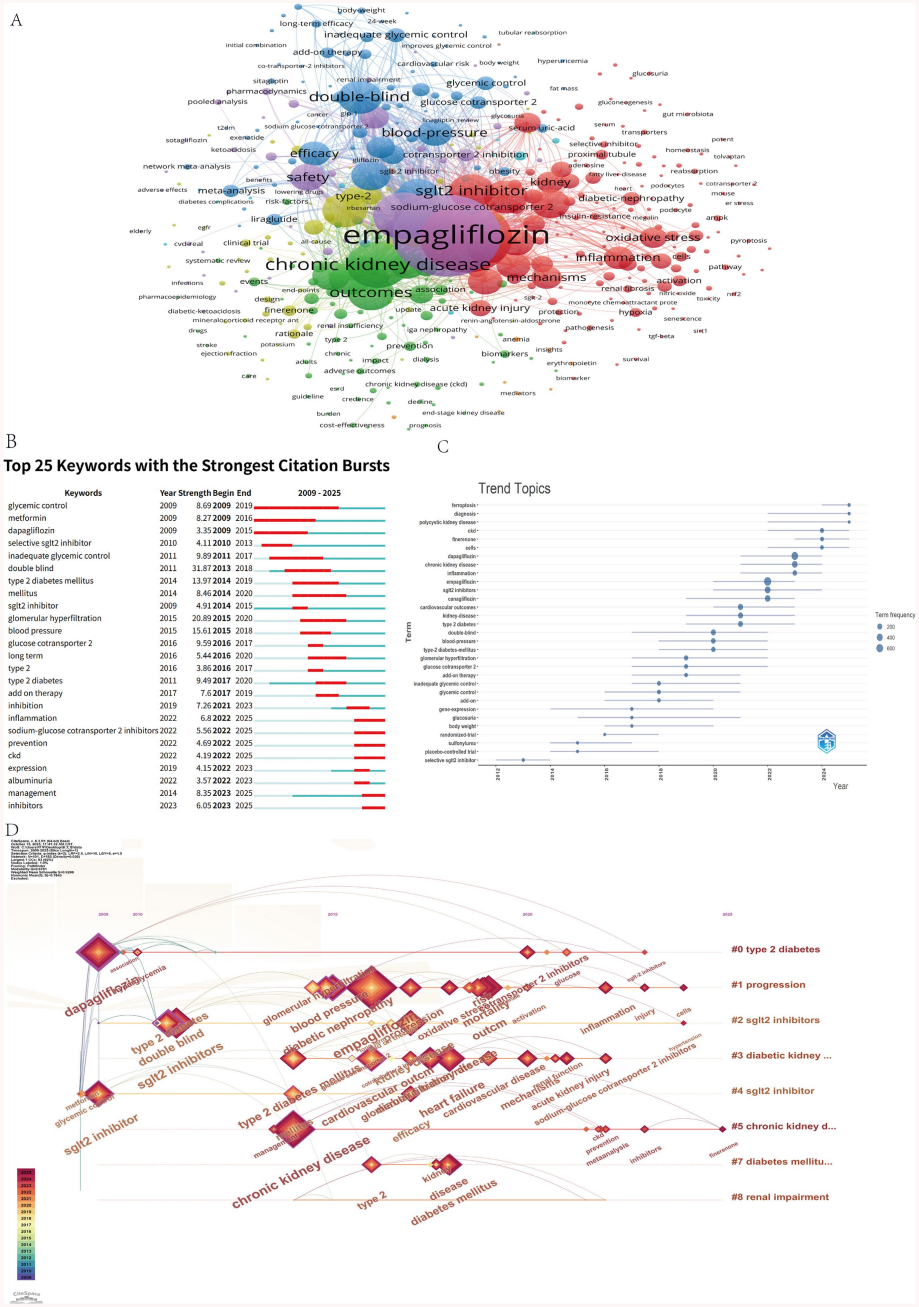
**(A)** Co-occurrence analysis of keywords. **(B)** Top 25 keywords with the strongest citation bursts. **(C)** Time zone change chart of keywords. **(D)** The timeline view of author’s keywords.

Figure 6A shows that the most frequently occurring keyword is empagliflozin, appearing 747 times. High-frequency keywords cluster into three main categories: drugs, diseases, and mechanisms/indicators. Drug-related terms include dapagliflozin and SGLT2 inhibitors; disease-related keywords encompass chronic kidney disease, kidney disease, and type 2 diabetes; mechanism- and outcome-related terms include cardiovascular outcomes, mortality, safety, inflammation, and oxidative stress. Citation burst analysis identifies temporal shifts in research focus across phases. From 2009 to 2017, research emphasis was primarily on glycemic control efficacy. Between 2018 and 2021, emerging keywords such as glomerular hyperfiltration and blood pressure indicate a shift toward understanding pathophysiological mechanisms and associated complications. From 2022 to 2025, the rise of terms like inflammation and management reflects an increasing focus on molecular mechanisms and clinical disease management strategies (Figure 6B). Keyword trend analysis (Figure 6C) reveals that early dominant terms such as glycemic control have declined in usage intensity. In contrast, terms including chronic kidney disease (CKD), cells, ferroptosis, and polycystic kidney disease have shown marked increases in frequency since 2022. As illustrated in Figure 6D, the overall research trajectory has evolved from a primary focus on glycemic control to broader themes of multi-organ protection, particularly emphasizing the renal protective effects of SGLT2 inhibitors. The target population has expanded beyond patients with type 2 diabetes to include individuals with chronic kidney disease regardless of diabetic status. Concurrently, mechanistic investigations have advanced to explore molecular pathways such as inflammation, oxidative stress, and ferroptosis, thereby strengthening the theoretical foundation for the clinical application of SGLT2 inhibitors.

### Clinical trial progress analysis

#### Advancing evidence-based clinical trial design

**Figure 7:**
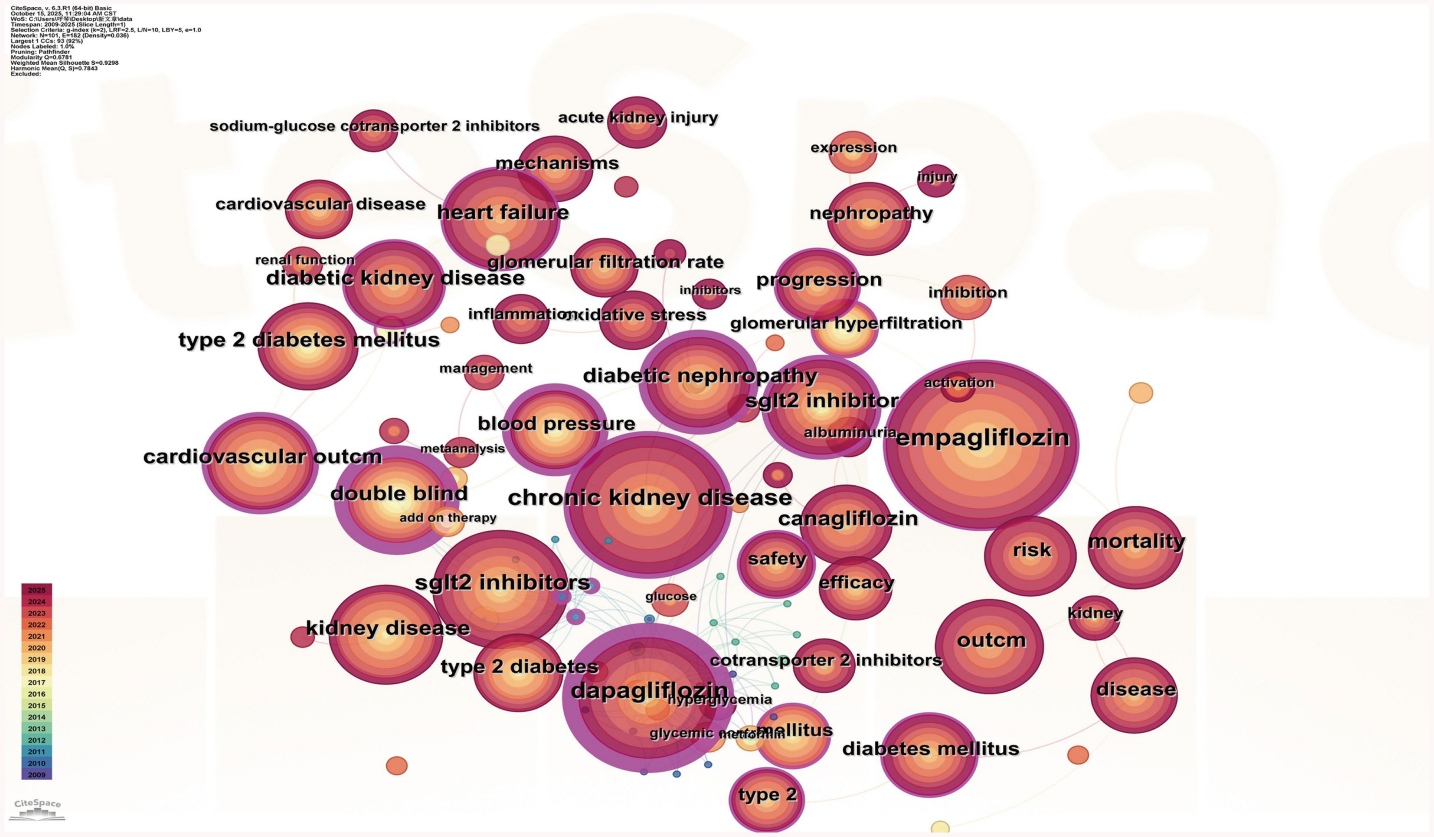
Co-occurrence of author’s keywords by using CiteSpace.

The terms “double blind” and “meta-analysis” occupy central positions in the co-occurrence map and are strongly associated with “chronic kidney disease,” indicating a strong emphasis on high-quality randomized controlled trial (RCT) design in this field (Figure 7). The prominence of double-blind methodology reflects its status as a cornerstone of methodological rigor in RCTs, while the frequent co-occurrence of meta-analysis underscores the importance placed on synthesizing evidence-based findings. This pattern is consistent with keyword co-occurrence trends observed in the WOSCC database, highlighting the critical role of robust clinical trial design in the application of SGLT2 inhibitors in nephrology. Outcome assessments predominantly focus on objective laboratory markers such as glomerular filtration rate, renal function, albuminuria and proteinuria. Keywords such as “risk,” “mortality,” and “efficacy” frequently co-occur, indicating a strong clinical focus on drug effects and associated risks, with insufficient attention to patient-centered endpoints like quality of life. Future research should incorporate validated nephrology-specific quality-of-life instruments, standardize trial procedures, and enhance transparency in data reporting.

#### Disease application spectrum centered on diabetic nephropathy and chronic kidney disease

The keywords “type 2 diabetes mellitus,” “diabetic nephropathy,” and “chronic kidney disease” form densely interconnected clusters in the co-occurrence network and exhibit strong associations with drug-related terms such as “SGLT2 inhibitors,” “empagliflozin,” and “canagliflozin.” This configuration clearly delineates the core therapeutic scope of SGLT2 inhibitor clinical trials. Starting from type 2 diabetes, the focus extends to diabetic nephropathy and encompasses the broader management of chronic kidney disease. Furthermore, their close linkage with “cardiovascular disease” and “heart failure” indicates that clinical investigations of these agents span multiple organ systems, reflecting a holistic therapeutic approach. Notably, the node corresponding to “acute kidney injury” remains relatively underdeveloped, while it exhibits an association with “SGLT2 inhibitors,” clinical trial focus remains notably less intense than that directed toward chronic kidney disease. This disparity suggests a gap between preclinical evidence and clinical translation, positioning acute kidney injury as a potential frontier for future clinical trial expansion.

#### Multidimensional integration: combination therapy and mechanistic synergy of SGLT2 inhibitors

The robust co-occurrence of “add-on therapy” with “chronic kidney disease” and “SGLT2 inhibitors,” along with its connection to mechanism-driven keywords such as “inflammation” and “oxidative stress,” indicates an ongoing effort to integrate SGLT2 inhibitors into comprehensive treatment strategies (Figure 7). These inhibitors are increasingly utilized as add-on therapies, combined either with established standard-of-care regimens for kidney disease or with anti-inflammatory and antioxidant interventions, aiming to achieve synergistic mechanisms that improve key outcomes, such as reducing albuminuria and slowing the decline in kidney function. Moreover, the robust associations between “SGLT2 inhibitors” and biomarkers like “glomerular filtration rate” and “albuminuria” reflect a multidimensional exploration linking pharmacological mechanisms to clinically relevant endpoints. Clinical trials now extend beyond efficacy evaluation to include the modulation of key pathological indicators in kidney disease. Moving forward, this integrative paradigm is expected to expand further, enabling the development of multi-target combination therapies and supporting more personalized management approaches for complex conditions such as chronic kidney disease.

## Discussion

### Basic information

This study presents the first comprehensive bibliometric analysis of research on SGLT2 inhibitors in kidney disease, based on publications indexed in the WOSCC from 2009 to 2025. A total of 1,561 relevant articles were identified, originating from 60 countries, 1,555 institutions, and involving 8,912 authors across 408 journals. The annual publication trend reveals three distinct developmental phases, each aligned with the release of pivotal clinical trials: EMPA-REG OUTCOME (2015), CREDENCE (2019), and DAPA-CKD (2020). Since 2022, annual output has consistently exceeded 230 publications, indicating sustained academic interest and reinforcing confidence for continued investment in both basic science and clinical research within this domain. From a national perspective, the United States has 267 papers with corresponding authors, recording the highest total citations and establishing itself as a central contributor in the field. China has 304 papers with corresponding authors (accounting for 19.5%), indicating deepening research in this field, but the average citations per paper (15) and MCP ratio (7.6%) are significantly lower than those of the United States (57.4 citations, 39.7%), suggesting that efforts need to continue in the depth of international collaboration. Although Australia has only 65 publications, it achieves an average of 217.6 citations per paper, demonstrating a notable impact per article. At the institutional level, the University of Toronto (278 publications), the University of Groningen (213 publications), and two multinational pharmaceutical companies (AstraZeneca and Boehringer Ingelheim) are among the top 10, with frequent collaboration, indicating that research on SGLT2 inhibitors has formed trends in clinical demand, mechanism validation, and drug repurposing.

Author co-occurrence analysis shows that Heerspink HJL from the University of Groningen is the most influential author in this field, with the highest number of publications and consistently high citations from 2016 to 2025. In terms of journals, *Diabetes Obesity & Metabolism* stands out with 100 publications, and the *New England Journal of Medicine* has a notable 7,640 local citations. Journals in this field are mostly focused on diabetes, nephrology, and cardiovascular and metabolic research, providing researchers with guidance for precisely selecting journals based on their findings.

The evolution of keywords has shifted from simply focusing on the glucose-lowering effects of SGLT2 inhibitors to the drugs’ mechanisms of action and multi-organ protection, such as for the kidneys. After 2022, terms like “ferroptosis, inflammation, polycystic kidney disease” have emerged, indicating that future hotspots for SGLT2 inhibitors may focus on cellular and molecular mechanisms, combination therapy interventions, and the treatment of non-diabetic kidney diseases.

### Research hotspots and frontiers

#### Expansion of treatment indications

SGLT2 inhibitors were initially developed as glucose-lowering agents for patients with type 2 diabetes mellitus (T2DM), intended as adjuncts to lifestyle interventions (17). As clinical evidence accumulated, their renoprotective effects in diabetic kidney disease (DKD) became increasingly evident. The EMPA-REG OUTCOME trial demonstrated that empagliflozin significantly reduces the risk of kidney disease progression (18). Similarly, the CANVAS program showed that canagliflozin slows albuminuria progression and improves renal outcomes in T2DM patients (19). These research findings provide robust evidence supporting the guideline recommendation that SGLT2 inhibitors may be used as a second-line or combination therapy in conjunction with RAS inhibitors in T2DM patients with proteinuric DKD. More recently, the therapeutic scope of SGLT2 inhibitors (SGLT2i) has expanded beyond diabetes to include non-diabetic chronic kidney disease (CKD), marking a significant frontier in nephrology. The DAPA-CKD trial was the first time confirmed that dapagliflozin significantly lowers the risk of major renal composite endpoints in patients with non-diabetic CKD (20). The trial-level estimates from DAPA-CKD were also used to calculate the benefits of combined RAS inhibitor and SGLT2 inhibitor therapy in patients with non-diabetic CKD (21). The EMPA-KIDNEY trial further confirmed the efficacy of empagliflozin in non-diabetic CKD, showing consistent renoprotection across varying levels of proteinuria and stages of kidney function (14). Building on the evidence from the aforementioned experimental studies, the 2023 KDIGO CKD guidelines recommend SGLT2 inhibitors as first-line kidney-protective agents in patients with CKD stages G2–G4 (eGFR ≥20 mL/min/1.73m^2^), irrespective of diabetes status or baseline UACR levels, thereby establishing their preeminence over other non-RAS inhibitor therapies. Collectively, these trials have catalyzed a paradigm shift in clinical practice, extending the use of SGLT2i from diabetic to non-diabetic forms of CKD.

#### Renal protective mechanisms

The renoprotective effects of SGLT2 inhibitors extend beyond their glucose-lowering properties and are mediated through multiple interconnected pathways, with tubuloglomerular feedback (TGF) regulation being a central mechanism. By suppressing the reabsorption of sodium and glucose in the proximal tubules, SGLT2 inhibitors augment sodium delivery to the distal tubules, thus triggering TGF activation. This subsequently leads to afferent arteriolar constriction, diminished intraglomerular pressure, and alleviation of glomerular hyperfiltration (22). Additionally, these agents contribute to slowing chronic kidney disease (CKD) progression by modulating intrarenal hemodynamics and improving endothelial function. Evidence indicates that SGLT2 inhibitors reduce arterial stiffness and enhance endothelium-dependent vasodilation, which collectively lower glomerular capillary wall pressure and decrease proteinuria (23). Overactivation of the sympathetic nervous system (SNS) is a recognized contributor to CKD progression (24). SGLT2 inhibitors indirectly suppress SNS activity by promoting natriuresis and reducing blood volume, thus mitigating the increased tubular sodium reabsorption and renal vasoconstriction induced by sympathetic activation, ultimately attenuating kidney injury (25, 26). At the cellular and molecular level, SGLT2 inhibitors can activate adenosine 5′-monophosphate (AMP)-activated protein kinase (AMPK) and inhibit the activity of the mammalian mechanistic target of rapamycin complex 1 (mTORC1), thereby promoting autophagy, clearing damaged organelles such as mitochondria, and alleviating renal tubular interstitial inflammation and fibrosis (27). Under hypoxic conditions, SGLT2 inhibitors regulate the expression of hypoxia-inducible factor (HIF) subtypes, inhibit HIF-1α expression, and improve renal inflammation and fibrosis; at the same time, they enhance HIF-2α activity, thereby improving the renal oxygen metabolic environment (28, 29).

It is noteworthy that the protective effects of SGLT2 inhibitors (SGLT2i) may vary across different types of CKD models. In diabetic nephropathy models, SGLT2i significantly reduce proteinuria and glomerulosclerosis by decreasing hyperglycemia-induced glomerular hyperfiltration (30). In contrast, in non-diabetic CKD models, protection is largely attributed to non-hemodynamic mechanisms, including anti-inflammatory, anti-fibrotic effects, and improved cellular energy metabolism (31). Moreover, environmental factors such as high dietary salt intake can impair TGF activation by SGLT2 inhibitors, potentially diminishing therapeutic efficacy—highlighting the influence of the local microenvironment on drug response (32). In summary, SGLT2 inhibitors confer renal protection via a multifaceted array of mechanisms, including modulation of TGF, improvement of tubular oxygenation, suppression of inflammation and fibrosis, induction of autophagy, and mitigation of oxidative stress. Future research should aim to delineate the relative contributions of these pathways in specific pathological contexts and support the development of personalized treatment strategies to maximize renoprotective outcomes.

#### Combination therapy strategies

As understanding of the renoprotective mechanisms of SGLT2 inhibitors (SGLT2i) deepens, research focus has shifted from monotherapy to multi-target combination approaches. Current efforts are increasingly directed toward evaluating SGLT2i in conjunction with other established or emerging renoprotective agents, particularly renin-angiotensin-aldosterone system inhibitors (RAASi), non-steroidal mineralocorticoid receptor antagonists (ns-MRAs), and endothelin receptor antagonists (ERAs). The CONFIDENCE trial is designed to assess the impact of combining empagliflozin with finerenone on proteinuria levels in patients with type 2 diabetes and chronic kidney disease (CKD), aiming to provide high-quality evidence for the synergistic potential of SGLT2i and ns-MRA co-administration (33). Similarly, the ZENITH-CKD trial investigates the combination of SGLT2 inhibitors with the ERA zibotentan, focusing on its ability to reduce proteinuria and slow the decline in kidney function (34). Studies demonstrate that combining SGLT2i with RAASi yields complementary and synergistic effects: SGLT2 inhibitors primarily reduce intraglomerular pressure and alleviate tubular metabolic stress, whereas RAASi further protect the kidney by blocking angiotensin II-mediated vasoconstriction and fibrotic signaling. Importantly, SGLT2i may also mitigate the risk of hyperkalemia associated with RAASi use, thereby improving the safety profile and enabling more sustained dual therapy (35). Regarding non-steroidal mineralocorticoid receptor antagonists (ns-MRAs), finerenone has been shown to significantly reduce the risk of major adverse cardiovascular events and kidney-related endpoints in patients with diabetes and CKD (36). Existing simulation studies predict that triple therapy combining SGLT2 inhibitors, glucagon-like peptide-1 (GLP-1) receptor agonists, and finerenone may reduce the 5-year risk of major adverse cardiovascular events (MACE) by 35% in 50-year-old patients with diabetes and proteinuria, while extending event-free survival by up to 3.2 years (37). Although large-scale Phase III clinical trials directly validating the long-term efficacy and safety of this combination regimen are still pending, emerging evidence from Phase II trials and *post-hoc* analyses supports its therapeutic potential. For instance, a *post-hoc* analysis of the AMPLITUDE-O trial demonstrated that dual therapy with GLP-1 receptor agonists and SGLT2 inhibitors further reduces the risk of heart failure hospitalization and lowers proteinuria levels, without increasing adverse event rates (38). Additionally, a network meta-analysis indicated that the combination of SGLT2 inhibitors (SGLT2i) and endothelin receptor antagonists (ERAs) is superior to monotherapy in reducing proteinuria, suggesting potential benefits in specific patient subgroups (39). In summary, combination therapies involving SGLT2i and other renoprotective agents are increasingly recognized as a pivotal direction in CKD management, with future research expected to generate more robust clinical evidence for multi-target treatment strategies.

### Limitations

Although this study provides a comprehensive bibliometric analysis of SGLT2 inhibitor research in kidney disease, offering valuable insights into the field’s evolution, several limitations should be acknowledged. First, the data were sourced exclusively from the WoSCC and Pubmed, which may result in the omission of relevant non-English publications or studies indexed in other major databases such as Scopus. While WoSCC is highly regarded for its rigorous indexing standards, integrating multiple databases in future studies could enhance data comprehensiveness and representativeness. Second, the study period was defined as January 1, 2009, to October 9, 2025; although this captures the key developmental phases of the field, the cutoff date may slightly affect the completeness of recent publication trends. Third, during data processing and visualization, potential biases could arise from inconsistent keyword indexing or inadequate handling of synonyms across different software platforms. Therefore, we performed data cleaning and preprocessing to minimize errors arising from the data.

## Conclusion

This study employed bibliometric methods to analyze 1,561 publications on SGLT2 inhibitors in kidney disease research indexed in the WoSCC between 2009 and 2025. The annual publication count shows a consistent upward trend. The United States and China emerged as leading contributors in terms of output, with institutions such as the University of Toronto and the University of Groningen playing central roles. International collaboration networks are predominantly coordinated by the United States. Key authors, including Heerspink HJL and Wheeler DC, have exerted substantial academic influence. Leading journals in the field include the *New England Journal of Medicine, Diabetes, Obesity and Metabolism, and Diabetes Care*. Research hotspots have evolved significantly: clinical indications have expanded from diabetic to non-diabetic CKD; mechanistic investigations have advanced from hemodynamic theories to molecular pathways involving inflammation, oxidative stress, and ferroptosis; and therapeutic approaches have shifted from monotherapy to multi-target combination strategies. Overall, SGLT2 inhibitors hold considerable promise in kidney disease research. This analysis enables researchers to rapidly grasp the current landscape, emerging trends, and frontiers in the field, informs future research directions, and provides a theoretical foundation for the rational clinical use of SGLT2 inhibitors.

## Data Availability

The original contributions presented in the study are included in the article/Supplementary material, further inquiries can be directed to the corresponding author.

## Glossary

ESRD: End-stage renal disease
SGLT2i: Sodium-glucose co-transporter 2 inhibitor
WoSCC: Web of Science Core Collection
ISN: International Society of Nephrology
CKD: Chronic kidney disease
DKD: Diabetic kidney disease
T2DM: Type 2 diabetes mellitus
AKI: Acute kidney injury
SCI-Expanded: Science Citation Index Expanded
SSCI: Social Sciences Citation Index
non-diabetic CKD: Non-diabetic chronic kidney disease
TGF: Tubuloglomerular feedback
SNS: Sympathetic nervous system
AMPK: Adenosine 5′-monophosphate AMP-activated protein kinase
mTORC1: Mechanistic target of rapamycin complex 1
HIF: Hypoxia-inducible factor
RAASi: Renin-angiotensin-aldosterone system inhibitor
ns-MRA: Non-steroidal mineralocorticoid receptor antagonist
ERA: Endothelin receptor antagonist
MACE: Major adverse cardiovascular events

## References

[1] Kovesdy CP. Epidemiology of chronic kidney disease: an update 2022. Kidney Int Suppl. (2022) 12:7–11. doi: 10.1016/j.kisu.2021.11.003, 35529086 PMC9073222

[2] GBD Chronic Kidney Disease Collaboration. Global, regional, and national burden of chronic kidney disease, 1990–2017: a systematic analysis for the Global Burden of Disease Study 2017. Lancet. (2020) 395:709–33. doi: 10.1016/S0140-6736(20)30045-3, 32061315 PMC7049905

[3] Afkarian M Zelnick LR Hall YN Heagerty PJ Tuttle K Weiss NS . Clinical manifestations of kidney disease among US adults with diabetes, 1988–2014. JAMA. (2016) 316:602–10. doi: 10.1001/jama.2016.10924, 27532915 PMC5444809

[4] Zhang L Zhao M-H Zuo L Wang Y Yu F Zhang H . China kidney disease network (CK-NET) 2016 annual data report. Kidney Int Suppl. (2020) 10:e97–e185. doi: 10.1016/j.kisu.2020.09.001, 33304640 PMC7716083

[5] Wright EM Loo DDF Hirayama BA. Biology of human sodium glucose transporters. Physiol Rev. (2011) 91:733–94. doi: 10.1152/physrev.00055.2009 21527736

[6] Romagnani P Agarwal R Chan JCN Levin A Kalyesubula R Karam S . Chronic kidney disease. Nat Rev Dis Primers. (2025) 11:8. doi: 10.1038/s41572-024-00589-9 39885176

[7] Bae J Won YJ Lee B-W. Non-albumin proteinuria (NAP) as a complementary marker for diabetic kidney disease (DKD). Life. (2021) 11:224. doi: 10.3390/life11030224, 33802211 PMC7998887

[8] Thomas MC Cherney DZI. The actions of SGLT2 inhibitors on metabolism, renal function and blood pressure. Diabetologia. (2018) 61:2098–107. doi: 10.1007/s00125-018-4669-0 30132034

[9] Huang W Chen Y-Y Li Z-Q He F-F Zhang C. Recent advances in the emerging therapeutic strategies for diabetic kidney diseases. Int J Mol Sci. (2022) 23:10882. doi: 10.3390/ijms231810882, 36142794 PMC9506036

[10] Perkovic V Jardine MJ Neal B Bompoint S Heerspink HJL Charytan DM . Canagliflozin and renal outcomes in type 2 diabetes and nephropathy. N Engl J Med. (2019) 380:2295–306. doi: 10.1056/NEJMoa1811744 30990260

[11] Heerspink HJL Stefánsson BV Correa-Rotter R Chertow GM Greene T Hou F-F . Dapagliflozin in patients with chronic kidney disease. N Engl J Med. (2020) 383:1436–46. doi: 10.1056/NEJMoa2024816, 32970396

[12] Zinman B Wanner C Lachin JM Fitchett D Bluhmki E Hantel S . Empagliflozin, cardiovascular outcomes, and mortality in type 2 diabetes. N Engl J Med. (2015) 373:2117–28. doi: 10.1056/NEJMoa1504720, 26378978

[13] Dai Z-C Chen J-X Zou R Liang X-B Tang J-X Yao C-W. Role and mechanisms of SGLT-2 inhibitors in the treatment of diabetic kidney disease. Front Immunol. (2023) 14:1213473. doi: 10.3389/fimmu.2023.1213473, 37809091 PMC10552262

[14] Herrington WG Staplin N Wanner C Green JB Hauske SJ Emberson JR . Empagliflozin in patients with chronic kidney disease. N Engl J Med. (2023) 388:117–27. doi: 10.1056/NEJMoa2204233, 36331190 PMC7614055

[15] Upadhyay A. SGLT2 inhibitors and kidney protection: mechanisms beyond tubuloglomerular feedback. Kidney360. (2024) 5:771–82. doi: 10.34067/KID.0000000000000425, 38523127 PMC11146657

[16] Thompson DF Walker CK. A descriptive and historical review of bibliometrics with applications to medical sciences. Pharmacotherapy. (2015) 35:551–9. doi: 10.1002/phar.1586 25940769

[17] Monami M Nardini C Mannucci E. Efficacy and safety of sodium glucose co-transport-2 inhibitors in type 2 diabetes: a meta-analysis of randomized clinical trials. Diabetes Obes Metab. (2014) 16:457–66. doi: 10.1111/dom.12244, 24320621

[18] Wanner C Inzucchi SE Lachin JM Fitchett D von Eynatten M Mattheus M . Empagliflozin and progression of kidney disease in type 2 diabetes. N Engl J Med. (2016) 375:323–34. doi: 10.1056/NEJMoa1515920 27299675

[19] Neal B Perkovic V Mahaffey KW de Zeeuw D Fulcher G Erondu N . Canagliflozin and cardiovascular and renal events in type 2 diabetes. N Engl J Med. (2017) 377:644–57. doi: 10.1056/NEJMoa1611925 28605608

[20] McEwan P Darlington O Miller R McMurray JJV Wheeler DC Heerspink HJL . Cost-effectiveness of dapagliflozin as a treatment for chronic kidney disease: a health-economic analysis of DAPA-CKD. Clin J Am Soc Nephrol. (2022) 17:1730–41. doi: 10.2215/CJN.03790322, 36323444 PMC9718008

[21] Vart P Vaduganathan M Jongs N Remuzzi G Wheeler DC Hou FF . Estimated lifetime benefit of combined RAAS and SGLT2 inhibitor therapy in patients with albuminuric CKD without diabetes. Clin J Am Soc Nephrol. (2022) 17:1754–62. doi: 10.2215/CJN.08900722, 36414316 PMC9718016

[22] Heerspink HJL Kosiborod M Inzucchi SE Cherney DZI. Renoprotective effects of sodium-glucose cotransporter-2 inhibitors. Kidney Int. (2018) 94:26–39. doi: 10.1016/j.kint.2017.12.027, 29735306

[23] Gaspari T Spizzo I Liu H Hu Y Simpson RW Widdop RE . Dapagliflozin attenuates human vascular endothelial cell activation and induces vasorelaxation: a potential mechanism for inhibition of atherogenesis. Diabetes Vasc Dis Res. (2018) 15:64–73. doi: 10.1177/1479164117733626 28976221

[24] Bell-Reuss E Trevino DL Gottschalk CW. Effect of renal sympathetic nerve stimulation on proximal water and sodium reabsorption. J Clin Invest. (1976) 57:1104–7. doi: 10.1172/JCI108355, 947953 PMC436757

[25] Wan N Fujisawa Y Kobara H Masaki T Nakano D Rahman A . Effects of an SGLT2 inhibitor on the salt sensitivity of blood pressure and sympathetic nerve activity in a nondiabetic rat model of chronic kidney disease. Hypertens Res. (2020) 43:492–9. doi: 10.1038/s41440-020-0410-8, 32060381

[26] Liu Y Wu M Xu B Kang L. Empagliflozin alleviates atherosclerosis progression by inhibiting inflammation and sympathetic activity in a normoglycemic mouse model. J Inflamm Res. (2021) 14:2277–87. doi: 10.2147/JIR.S309427, 34103961 PMC8180283

[27] Packer M. SGLT2 inhibitors produce cardiorenal benefits by promoting adaptive cellular reprogramming to induce a state of fasting mimicry: a paradigm shift in understanding their mechanism of action. Diabetes Care. (2020) 43:508–11. doi: 10.2337/dci19-0074, 32079684

[28] Chen R Xu M Hogg RT Li J Little B Gerard RD . The acetylase/deacetylase couple CREB-binding protein/sirtuin 1 controls hypoxia-inducible factor 2 signaling. J Biol Chem. (2012) 287:30800–11. doi: 10.1074/jbc.M111.244780, 22807441 PMC3436323

[29] Bessho R Takiyama Y Takiyama T Kitsunai H Takeda Y Sakagami H . Hypoxia-inducible factor-1α is the therapeutic target of the SGLT2 inhibitor for diabetic nephropathy. Sci Rep. (2019) 9:14754. doi: 10.1038/s41598-019-51343-1, 31611596 PMC6791873

[30] Vallon V Gerasimova M Rose MA Masuda T Satriano J Mayoux E . SGLT2 inhibitor empagliflozin reduces renal growth and albuminuria in proportion to hyperglycemia and prevents glomerular hyperfiltration in diabetic Akita mice. Am J Physiol Renal Physiol. (2014) 306:F194–204. doi: 10.1152/ajprenal.00520.2013, 24226524 PMC3920018

[31] Zeng S Delic D Chu C Xiong Y Luo T Chen X . Antifibrotic effects of low dose SGLT2 inhibition with empagliflozin in comparison to Ang II receptor blockade with telmisartan in 5/6 nephrectomised rats on high salt diet. Biomed Pharmacother. (2022) 146:112606. doi: 10.1016/j.biopha.2021.112606, 34968924

[32] Tauber P Sinha F Berger RS Gronwald W Dettmer K Kuhn M . Empagliflozin reduces renal hyperfiltration in response to uninephrectomy, but is not nephroprotective in UNx/DOCA/salt mouse models. Front Pharmacol. (2021) 12:761855. doi: 10.3389/fphar.2021.761855, 34992532 PMC8724563

[33] Green JB Mottl AK Bakris G Heerspink HJL Mann JFE McGill JB . Design of the COmbinatioN effect of FInerenone anD EmpaglifloziN in participants with chronic kidney disease and type 2 diabetes using a UACR Endpoint study (CONFIDENCE). Nephrol Dial Transplant. (2023) 38:894–903. doi: 10.1093/ndt/gfac198, 35700142 PMC10064838

[34] Heerspink HJL Kiyosue A Wheeler DC Lin M Wijkmark E Carlson G . Zibotentan in combination with dapagliflozin compared with dapagliflozin in patients with chronic kidney disease (ZENITH-CKD): a multicentre, randomised, active-controlled, phase 2b, clinical trial. Lancet. (2023) 402:2004–17. doi: 10.1016/S0140-6736(23)02230-4, 37931629

[35] Neuen BL Oshima M Agarwal R Arnott C Cherney DZ Edwards R . Sodium-glucose cotransporter 2 inhibitors and risk of hyperkalemia in people with type 2 diabetes: a meta-analysis of individual participant data from randomized, controlled trials. Circulation. (2022) 145:1460–70. doi: 10.1161/CIRCULATIONAHA.121.057736, 35394821

[36] Agarwal R Filippatos G Pitt B Anker SD Rossing P Joseph A . Cardiovascular and kidney outcomes with finerenone in patients with type 2 diabetes and chronic kidney disease: the FIDELITY pooled analysis. Eur Heart J. (2022) 43:474–84. doi: 10.1093/eurheartj/ehab777, 35023547 PMC8830527

[37] Neuen BL Heerspink HJL Vart P Claggett BL Fletcher RA Arnott C . Estimated lifetime cardiovascular, kidney, and mortality benefits of combination treatment with SGLT2 inhibitors, GLP-1 receptor agonists, and nonsteroidal MRA compared with conventional care in patients with type 2 diabetes and albuminuria. Circulation. (2024) 149:450–62. doi: 10.1161/CIRCULATIONAHA.123.067584, 37952217

[38] Lam CSP Ramasundarahettige C Branch KRH Sattar N Rosenstock J Pratley R . Efpeglenatide and clinical outcomes with and without concomitant sodium-glucose cotransporter-2 inhibition use in type 2 diabetes: exploratory analysis of the AMPLITUDE-O trial. Circulation. (2022) 145:565–74. doi: 10.1161/CIRCULATIONAHA.121.057934 34775781

[39] Yamada T Wakabayashi M Bhalla A Chopra N Miyashita H Mikami T . Cardiovascular and renal outcomes with SGLT-2 inhibitors versus GLP-1 receptor agonists in patients with type 2 diabetes mellitus and chronic kidney disease: a systematic review and network meta-analysis. Cardiovasc Diabetol. (2021) 20:14. doi: 10.1186/s12933-020-01197-z, 33413348 PMC7792332

