# Supplementary material for: Application of SGLT2 inhibitors in kidney diseases: a bibliometric analysis
Source: Front Med (Lausanne). 2026 Feb 6;13:1768225. doi: 10.3389/fmed.2026.1768225 (PMC12920433; doi:10.3389/fmed.2026.1768225)
Supplement: Supplementary file 1 [file Table_1.docx]

**Web of Science Core Collection (WoSCC)**

(TS = (“SGLT2 inhibit” OR “sodium–glucose cotransporter 2 inhibit” OR “sodium–glucose cotransporter type 2 inhibit” OR “sodium–glucose linked transporter type 2inhibit” OR “sodium glucose cotransporter 2 inhibit” OR “sodium/glucose cotransporter 2 inhibit” OR “sodium–glucose co-transporter 2 inhibit” OR “sodium–glucose transporter 2 inhibit” OR “sodium glucose co-transporter 2 inhibit” OR “sodium–glucose transport protein 2 inhibit” OR “sodiumdependent glucose cotransporter 2 inhibit” OR “sodium–glucose contransporter proteins 2 inhibit” OR “sodium-dependent glucose transporters 2 inhibit” OR “sodium glucose transport protein 2inhibit” OR “gliflozin” OR “canagliflozin” OR “dapagliflozin” OR “empagliflozin” OR “ertugliflozin” OR “ipragliflozin” OR “luseogliflozin” OR “tofogliflozin”))AND(TS= ("kidney disease" OR "renal disorder"OR"kidney physiology"OR "chronic kidney disease" OR "diabetic nephropathy" OR “Diabetic Kidney Disease”OR"hypertensive nephropathy"OR"renal fibrosis"OR"kidney fibrosis"OR"renal interstitial fibrosis"OR"kidney interstitial fibrosis"OR"renal tubulointerstitial fibrosis"OR"kidney tubulointerstitial fibrosis"OR"glomerular fibrosis"OR"glomerulosclerosis"O R"glomerular sclerosis"OR"renal vascular fibrosis"OR"kidney fibrosis"OR"focal segmental glomerulosclerosis" OR"kidney injury"OR"acute kidney injury" OR"kidney histology"OR"diabetic nephropathy"OR"hypertensive nephropathy" OR"glomerulonephritis" OR"polycystic kidney disease"OR“glomeruli”OR"tubular"OR"renal tubular dysfuction"OR"nephrotoxicity"))

**PubMed**

((("Sodium-Glucose Transporter 2"[Mesh] OR"Sodium-Glucose Cotransporter 2 Inhibitors"[Mesh] OR "sglt2 inhibitor"[tiab] OR "sglt-2 inhibitor"[tiab] OR"SGLT2"[tiab] OR"gliflozin"[tiab] OR canagliflozin[tiab] OR dapagliflozin[tiab] OR empagliflozin[tiab] OR ertugliflozin[tiab] OR ipragliflozin[tiab] OR luseogliflozin[tiab] OR tofogliflozin[tiab] OR "sodium glucose cotransporter 2 inhibitor"[tiab]) )AND (("Kidney Diseases"[Mesh] OR"Renal Insufficiency, Chronic"[Mesh] OR"Diabetic Nephropathies"[Mesh] OR "Glomerulosclerosis, Focal Segmental"[Mesh] OR "Acute Kidney Injury"[Mesh] OR "kidney disease"[tiab] OR "renal disorder"[tiab] OR "chronic kidney disease"[tiab] OR "diabetic nephropathy"[tiab] OR "diabetic kidney disease"[tiab] OR "hypertensive nephropathy"[tiab] OR "renal fibrosis"[tiab] OR"kidney fibrosis"[tiab] OR "renal interstitial fibrosis"[tiab] OR "glomerulosclerosis"[tiab] OR"focal segmental glomerulosclerosis"[tiab] OR "acute kidney injury"[tiab] OR"kidney injury"[tiab] OR"glomerulonephritis"[tiab] OR "polycystic kidney disease"[tiab] OR"renal tubular dysfunction"[tiab] OR "nephrotoxicity"[tiab]) ))AND ("2009/01/01"[pdat] : "2025/10/09"[pdat])
